# Research on the Drivers of Entrepreneurship Education Performance of Medical Students in the Digital Age

**DOI:** 10.3389/fpsyg.2021.733301

**Published:** 2021-10-29

**Authors:** Zehai Long, Guojing Zhao, Jing Wang, Mengting Zhang, Shaoyu Zhou, Ling Zhang, Zhaoxin Huang

**Affiliations:** ^1^Institute of China Innovation and Entrepreneurship Education, Wenzhou Medical University, Wenzhou, China; ^2^School of Education, Hangzhou Normal University, Hangzhou, China

**Keywords:** medical students, entrepreneurship education performance, driving factors, digital economy, ridge regression model

## Abstract

COVID-19 has made the entire society pay more attention to medical students training. Medicine development is inseparable from the spirit of innovation, focusing on cultivating medical students' innovative awareness and improving entrepreneurship education performance, which has an irreplaceable effect on both the students themselves and the society. This study is based on the ridge regression model to study the driving factors of the entrepreneurship education performance of medical students. Compared with traditional multiple regression, it can improve the consistency of parameter estimation and obtain more realistic results. Based on a large sample of empirical survey data of 24,677 medical students in China, this study analyzed the driving factors of the entrepreneurship education performance of medical students and found that medical students of different genders have differences in entrepreneurship education performance; the digital economy impacts entrepreneurship education performance of medical students; entrepreneurship course, entrepreneurship faculty, entrepreneurship competition, entrepreneurship practice, and entrepreneurship policy have a driving effect on the entrepreneurship education performance of medical students. Meanwhile, the impact of entrepreneurship policy is the most obvious, followed by entrepreneurship practice and entrepreneurship competition, followed by entrepreneurship course and entrepreneurship faculty.

## Introduction

Swept by the global COVID-19 pandemic, the world's major economies have suffered an unprecedented impact, with a decline in economic levels and a significant rise in unemployment. For example, in April 2020, the unemployment rate according to the Organization for Economic Co-operation and Development was 8.7249% and the Group of Seven as high as 9.0402%. According to the relevant data released by the International Labour Organization on April 7, 2020, the COVID-19 epidemic has affected 81% of the world's workers. In China, the impact of the COVID-19 epidemic cannot be underestimated. Many enterprises have delayed opening or even closed, while the number of college graduates has continued to rise to new highs. In this worldwide situation, employment prospects are bleak. Entrepreneurship education plays an important role in alleviating pressure on employment. On the one hand, entrepreneurship education can enhance college students' entrepreneurial awareness and entrepreneurial ability and can help achieve independent entrepreneurship. On the other hand, more college students may not start their own companies, and implementing entrepreneurship education can enable them to create new careers with “entrepreneurial mentality and innovative thinking” in their future jobs, and improve the level and quality of employment. This is precisely in line with UNESCO's definition of entrepreneurship education, which states that “entrepreneurship education” refers to the cultivation of pioneering individuals in a broad sense, which is equally important for salaried people. Based on entrepreneurship education importance, research on entrepreneurship education has had a high uptake and has made significant progress (Kuratko, 2005; Neck and Greene, 2011; Wang et al., 2019).

However, for medical students' entrepreneurship education, both teaching practice and academic research are still very scarce (Ozdemir et al., 2019). The British Higher Education Entrepreneurship Survey report points out that entrepreneurship education in areas such as medicine accounts for the least (1%). Is it really because the entrepreneurship education of medical students is not important? The answer is obviously not. Medical education is the cornerstone of the development of medical and health causes, and the development of medicine is inseparable from the spirit of innovation. Paying attention to cultivating medical students' innovative consciousness and improving their innovative ability plays an irreplaceable role not only for the students themselves but also for the whole society. Some studies have shown that entrepreneurship education plays an important role in promoting the innovative spirit and ability of medical students and also helps improve their employability (Li, 2017). However, because of the professionalism and practicality of medicine, medical students have heavy learning tasks and limited time. They lack enthusiasm for innovation and entrepreneurship, and many medical students have no entrepreneurial consciousness, let alone entrepreneurial spirit. There are still several problems in implementing entrepreneurship education in medical colleges and universities because of the lack of traditional education. First, many medical colleges and universities do not pay enough attention to entrepreneurship education, and the concept of entrepreneurship education is not deep into the teaching system. Second, entrepreneurship education is separated from professional education. Finally, the faculty of entrepreneurship education is very weak. Consequently, the entrepreneurship education performance of medical students is not high. With the reform and development of the medical and health field and the rise of medical and health entrepreneurship represented by the large health industry, the demand for new customized services is increasing, and the entrepreneurial spirit is on the rise, creating more entrepreneurial opportunities (Wilson et al., 2012) for the participants in the health care system. Many doctors pay more attention to improving the service environment, and they have more entrepreneurial advantages (Callaway and Dobrzykowski, 2009). Many countries are paying increasing attention to medical entrepreneurship. In the UK, the government supports the transformation of new technologies for medical innovation and entrepreneurship, such as MR scanning and protein and DNA sequencing. Japan provides a variety of facilities for entrepreneurship, especially in the areas of elderly patient care, infant care, and other areas to implement more preferential policies to support it. Especially in the digital age, digital technology application and the development of the digital industry help to reduce entrepreneurial barriers, integrate entrepreneurial resources, and facilitate entrepreneurship (Fossen and Sorgner, 2021). A series of new fields, such as online medical communities and Internet hospitals, have brought more opportunities for medical students to start businesses. Therefore, how to effectively improve the entrepreneurship education performance of medical students has become a top priority, but research on the entrepreneurship education performance of medical students is not extensive, and empirical research is rare. Considering gender differences and the impact of digital age, this study theoretically attempts to explore the impact of gender, the digital economy, entrepreneurship course, entrepreneurship faculty, entrepreneurship competition, entrepreneurship practice, and entrepreneurship policy on entrepreneurship education performance. Based on the data of 24,677 questionnaires, ridge regression was used to conduct empirical research to answer the following questions:

Is there any difference in the entrepreneurship education performance of medical students of different genders?
Does the digital economy have an impact on the entrepreneurship education performance of medical students?
Do entrepreneurship course, entrepreneurship faculty, entrepreneurship competition, entrepreneurship practice, and entrepreneurship policy drive the entrepreneurship education performance of medical students, and which factors drive it more?

The remainder of this study is organized as follows. The next section discusses the factors that influence the entrepreneurship education performance of medical students as the basis for our hypotheses. Section Research Design and Theoretical Model explains the data sources, variable measures, and the ridge regression models used in this study. Section Analysis of the Research Process and Results describes the empirical process and analysis results. It concludes with a discussion, conclusions, implications for theory, implications for practice and limitations, and further research opportunities.

## Literature Review and Research Hypotheses

### Gender and Entrepreneurship Education Performance

There is a significant difference between female and male entrepreneurs (DeMartino and Barbato, 2002; Maes et al., 2014). Entrepreneurial activities have high risk and high uncertainty and are generally considered being male-dominated areas. The Global Entrepreneurship Monitoring report 2020/2021 (GEM, 2021) points out that the number of male entrepreneurs is higher than female entrepreneurs worldwide. The research also shows that female college students lag behind male college students in becoming entrepreneurs (Hsu et al., 2007). This underrepresentation of women in entrepreneurship may be because of gender-related restrictions (Hsu et al., 2007)—called the “Pipeline effect” (Wilson et al., 2007). Such restrictions hinder women development. In starting a business, women may think that they have the same entrepreneurial ability as men. Paradoxically, they also think that because the entrepreneurial environment of women groups makes it more difficult for women to start a business than men, they get less in return (Zhang et al., 2014). Additionally, in the Global Entrepreneurship Monitoring report 2020/2021 (2021), women from six economies, including Central and East Asia, had higher levels of entrepreneurship than men in 2020. In the health care field, it often involves taking care of others, and this kind of work usually belongs to women (Hechavarría et al., 2017). Women are more likely to care about others and are more humane—giving them an advantage. Entrepreneurship in the healthcare field focuses not only on business value but also on social value. Women are more likely to become social entrepreneurs (Dickel and Eckardt, 2021). From this level, female medical students are likely to achieve higher performance in entrepreneurship education. Based on the above analysis, we propose hypothesis 1.

*Hypothesis 1: Gender significantly affects the entrepreneurship education performance of medical students*.

### Digital Economy and Entrepreneurship Education Performance

The digital economy is booming, especially in developing countries (Bukht and Heeks, 2017). The digital economy has brought changes to entrepreneurial activities and processes (Srinivasan and Venkatraman, 2018), and is constantly reshaping the market and society, which will have a series of effects on innovation, entrepreneurship, and the risk creation process (Giones and Brem, 2017). In this context, Internet use has significantly increased the probability of entrepreneurship (Mack et al., 2017). The information, communications, and technology (ICT) sector, which produces basic digital products and services, is the core “digital sector” (Bukht and Heeks, 2017) of the digital economy. Better use of ICT helps to generate, integrate, develop, and enhance critical resources to create innovative business opportunities and gain competitive advantage (Yunis et al., 2018). New digital technologies (big data, mobile, social media, cloud solutions, etc.) have spawned new ways of collaboration, based on open system standards and open sharing technologies, bringing a series of new opportunities with great potential business value, and greatly reducing startup costs (Zhao et al., 2015). The development of the digital economy lowers the threshold of entrepreneurship and optimizes entrepreneurial resources allocation—helping to improve students' entrepreneurial willingness, enhance their entrepreneurial skills, and improve entrepreneurship education performance. Based on this, Hypothesis 2 is proposed.

*Hypothesis 2: The digital economy has a significant positive impact on the entrepreneurship education performance of medical students*.

### Entrepreneurship Course and Entrepreneurship Education Performance

The entrepreneurship course includes information on how students identify and shape opportunities, evaluate business concepts, develop business plans, fund and start businesses, develop new businesses, and case studies that should be discussed in class, providing students with another opportunity to test entrepreneurial strategies and understand the successes and failures of new businesses (Moses and Akinbode, 2014). The school offers many courses and programs related to entrepreneurship, which are designed to provide students with motivation and confidence to start a business (Fayolle et al., 2006) and enhance their willingness to start a business (Tkachev and Kolvereid, 1999; Mueller, 2011). A well-established entrepreneurship course and experience is a long way to improve students' knowledge and skills (Byun et al., 2018). Chen et al. (2015) adopted the experimental design of a single-group pre-test and post-test, taking 41 college students who took the course of innovation and entrepreneurship management as subjects. After 18 weeks of teaching, it was found that the students were highly satisfied with the course design and teaching, and their entrepreneurial knowledge and skills improved. Based on this, Hypothesis 3 is proposed.

*Hypothesis 3: Entrepreneurship course has a significant positive impact on the entrepreneurship education performance of medical students*.

### Entrepreneurship Faculty and Entrepreneurship Education Performance

Teachers play a very important role in entrepreneurship education, and their leadership is very important for the cultivation of students' entrepreneurial ability, and their understanding of entrepreneurship education will affect entrepreneurship education performance. Seikkula-Leino et al. (2010) believes that entrepreneurship education should strengthen the teachers' importance and develop teachers' learning from the self-reflection perspective, to promote students' entrepreneurial knowledge and skills. Teachers' abilities are important for entrepreneurship education (Huang et al., 2020) and play an important role in entrepreneurship education (Teerijoki and Murdock, 2014; Ruskovaara and Pihkala, 2015). Through a series of structured activities, teachers can encourage students to learn how to start a business and strengthen entrepreneurial practices (Fejes et al., 2019). Based on the above analysis, we propose hypothesis 4.

*Hypothesis 4: Entrepreneurship faculty has a significant positive impact on the entrepreneurship education performance of medical students*.

### Entrepreneurship Competition and Entrepreneurship Education Performance

The entrepreneurship competition emphasizes knowledge and practice integration, playing an important role in creating jobs, reducing unemployment rates, and improving entrepreneurship education performance. Through the entrepreneurship competition, students can learn what elements and knowledge a complete business plan should include, and it also provides students with a favorable opportunity to practice entrepreneurship (Chang and Sung, 2009). Entrepreneurship competition encourages individuals to start their businesses and stimulate entrepreneurship and innovation genes (Yan et al., 2018). Entrepreneurship competition can cultivate students' innovative thinking that belongs to a kind of intensive entrepreneurship education (Hasan et al., 2017). Based on this, Hypothesis 5 is proposed.

*Hypothesis 5: Entrepreneurship competition has a significant positive impact on the entrepreneurship education performance of medical students*.

### Entrepreneurship Practice and Entrepreneurship Education Performance

Entrepreneurship practice is an effective extension and enrichment of classroom teaching in entrepreneurship education in colleges and universities (Wang, 2020). Higgins et al. (2018) believe that through practical learning, students can better apply their knowledge and skills to real entrepreneurship, and it is very important for students to gain experience through entrepreneurial practice. Entrepreneurship requires practice, and practice-based teaching methods include entrepreneurship as a course assignment, entrepreneurial games, and computer simulations to produce a high-quality learning experience, which can promote knowledge “beyond the analytical skills development and promote the confidence and motivation of contemporary entrepreneurship education” (Neck and Greene, 2011). For example, the Social Practical Wisdom curriculum is based on practical wisdom, focusing on integrating it into practice, creating comprehensive and rich social practice programs, developing students' knowledge and skills, and ultimately achieving positive long-term social change and solving social problems (Zhu et al., 2016). Based on this, Hypothesis 6 is proposed.

*Hypothesis 6: Entrepreneurship practice has a significant positive impact on the entrepreneurship education performance of medical students*.

### Entrepreneurship Policy and Entrepreneurship Education Performance

As a way to achieve significant economic benefits, government policies are increasingly inclined to promote entrepreneurship (O'Connor, 2013). Therefore, the government tries to use entrepreneurship education to stimulate a higher level of economic activity. Some countries have implemented policies to promote entrepreneurship education. Sweden launched an official entrepreneurship strategy in education and changed its curriculum in 2011 so that all students from pre-school to grade 12 should learn about entrepreneurship, rather than limiting the subject to business schools and higher education. In 2015, Chinese Premier Li Keqiang emphasized in his government report that “mass entrepreneurship and innovation” is one of the “twin engines” driving China's economic development. Many universities prioritize entrepreneurship education programs (Lin and Xu, 2017). Resultantly, the government has increased funding and launched new programs to promote entrepreneurship education (Hoppe, 2016) in colleges and universities. Based on this, Hypothesis 7 is proposed.

*Hypothesis 7: Entrepreneurship policy has a significant positive impact on the entrepreneurship education performance of medical students*.

**Figure 1:**
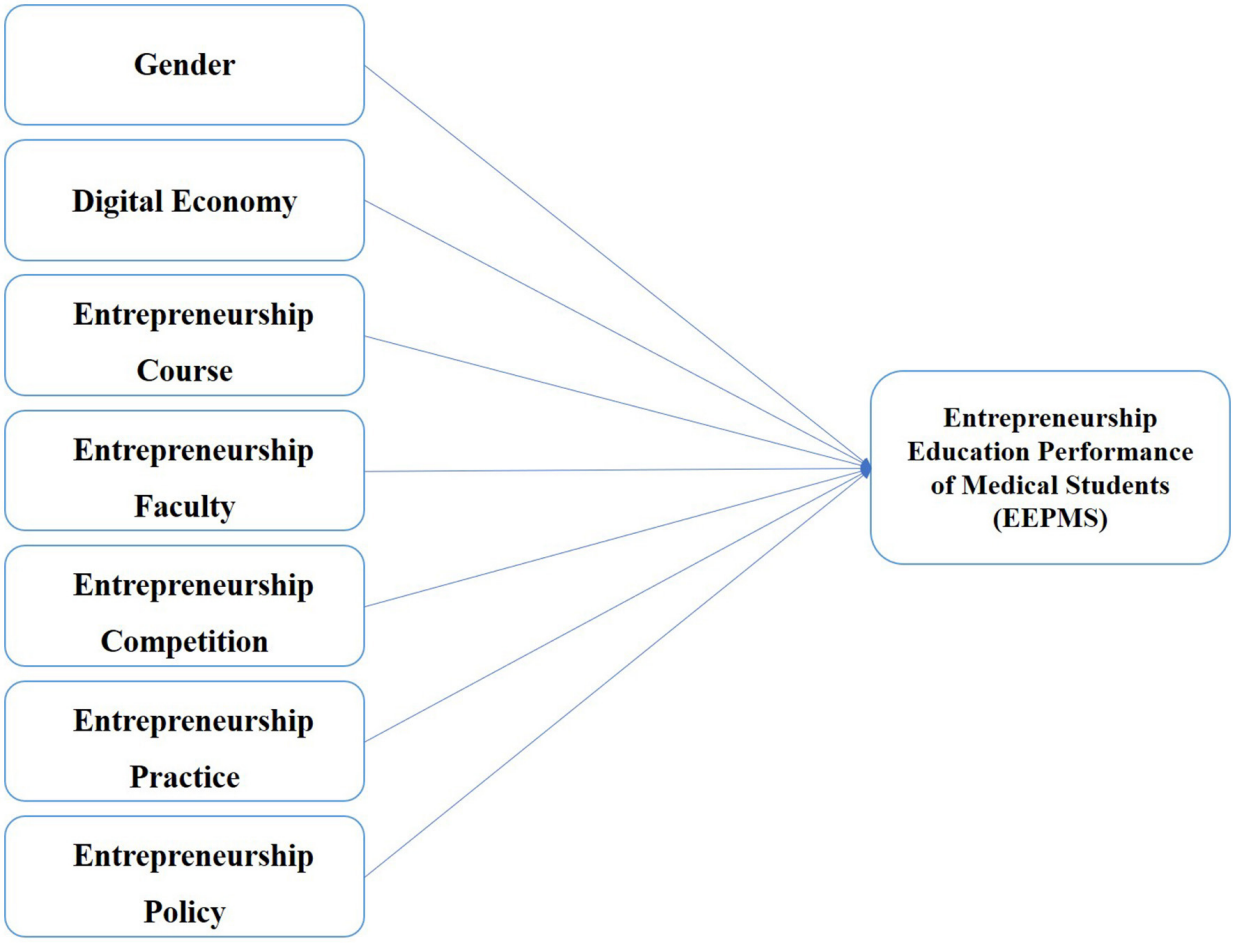
Research framework of EEPMS.

According to the above seven hypotheses, a model of the drivers of entrepreneurship education performance of medical students (EEPMS) in the digital era is developed, as shown in Figure 1.

## Research Design and Theoretical Model

### Questionnaire Design

Based on the existing relevant researches and literatures, the questionnaire is constantly revised and designed after several rounds of expert discussion and data testing. Our research team conducted research randomly using questionnaires and interviews with teachers involved in innovation and entrepreneurship education, as well as students and graduates who had received innovation and entrepreneurship education in 1,231 universities in 31 provinces (autonomous regions and municipalities directly under the central government) of China. 201,034 questionnaires, 283 interview records of over 500,000 words, of which 187,914 questionnaires were received in the Student Paper, after excluding 17,150 invalid questionnaires caused by short response time and invalid school names, 170,764 valid questionnaires were obtained, accounting for 90.87%, of which 24,677 medical students were screened out. The questionnaires covered basic background information of students and entrepreneurship course, entrepreneurship faculty, entrepreneurship competition, entrepreneurship practice, entrepreneurship policy, and entrepreneurship education performance scales, on a five-point Likert scale, with five representing strongly agree, 4 representing relatively agree, three representing average, two representing relatively disagree, and one representing strongly disagree.

### Research Variables System Construction and Data Sources

Fayolle et al. (2006) uses planned behavior theory and realizes that entrepreneurship education is not only understood as an opinion to start new business but as a method to change students' attitudes and values from the concept, so that they have a stronger entrepreneurial will and spirit. Matlay (2006) believes that entrepreneurship education improves the quality and quantity of entrepreneurs by influencing entrepreneurial attitudes, knowledge, and skills. The Chinese Ministry of Education, in “Opinions on Promoting Innovation and Entrepreneurship Education in Higher Education and Student Entrepreneurship,” pointed out that the core of entrepreneurship education is to enhance students' innovation, entrepreneurial awareness, and entrepreneurial ability. This study measures the EEPMS by enriching entrepreneurial knowledge, cultivating innovation spirit, improving entrepreneurial skills, stimulating entrepreneurial intentions, and overall satisfaction.

The factors affecting the EEPMS are mainly studied in terms of gender, digital economy, entrepreneurship course, entrepreneurship faculty, entrepreneurship competition, entrepreneurship practice, and entrepreneurship policy. Gender was set as a dummy variable with “1 for male” and “0” for female. The digital economy was adopted from the digital economy development index published by the China Electronics Information Industry Development Institute. The China Electronics Information Industry Development Research Institute is a research institution directly under the Ministry of Industry and Information Technology of China, also known as the Saedi Research Institute. The digital economy development index is composed of four primary indicators, 10 secondary indicators, and 38 tertiary indicators, including basic indicators, industrial indicators, integration indicators, and environmental indicators. The Chinese average value of the digital economy development index is 32.0, with 11 provinces and cities above the average value. The entrepreneurship course, entrepreneurship faculty, entrepreneurship competition, entrepreneurship practice, and entrepreneurship policy are all taken from the questionnaire. Entrepreneurship course is measured by three items (Nichols and Armstrong, 2003; Byun et al., 2018): “Diverse types of entrepreneurship education course,” “The content of the entrepreneurship course is closely integrated with your own professional knowledge,” “The content of the entrepreneurship course is closely aligned with the cutting-edge trends of the times.” Entrepreneurship faculty is measured by three items (Cheung, 2008; Seikkula-Leino et al., 2015): “Teachers teach a variety of styles,” “Teachers with entrepreneurial experience,” and “Teachers with extensive experience in teaching entrepreneurship education.” Entrepreneurship competition is measured by three items (Hasan et al., 2017; Watson and McGowan, 2019): “Variety of entrepreneurship competition,” “Entrepreneurship competition projects entered are more likely to be landed,” and “High degree of integration of entrepreneurship competition projects with the profession.” Entrepreneurship practice is measured by six items (Fan et al., 2013; Higgins et al., 2018): “Entrepreneurship practice with on and off-campus mentors,” “Entrepreneurship practice is supported by a dedicated start-up fund,” “The school offers an integrated entrepreneurship practice service,” “There is an independent college students pioneer park for entrepreneurship practice,” “Dedicated off-campus practice base for entrepreneurship practice,” and “High degree of integration of practical entrepreneurship projects with professional studies.” Entrepreneurship policy is measured by four items (Douglas and Shepherd, 2002; Audretsch, 2015): “State tax relief for university students starting their own businesses,” “Local governments simplify the application process for university student business registration,” “The university provides a start-up fund (interest-free loan) for starting a business,” and “Free training from the community to guide your business.”

The digital economy was adopted from the digital economy development index published by the China Electronics Information Industry Development Institute. The other variables (gender, entrepreneurship course, entrepreneurship faculty, entrepreneurship competition, entrepreneurship practice, entrepreneurship policy, and entrepreneurship education performance of medical students) are all taken from the questionnaire. Datas were nested analyzed with the approach of mixed embeddedness (Deng et al., 2020; Brieger and Gielnik, 2021).

### Ridge Regression Model

The ridge regression model was proposed by Hoerl (1962). Subsequently, Hoerl and Kennard (1970a,b) explored in detail and used a modified least squares method for solving independent variable multi-collinearity in linear regression analysis.

The general multiple linear regression model is:


(1) Y=α+Xβ+μ,

of which


(2) $$\text{Y}={\left(\begin{array}{c} Y_{1}\\ Y_{2}\\ \vdots \\ Y_{n} \end{array}\right)}_{n\times 1}\text{X}={\left[\begin{array}{cccc} X_{11} & X_{12} & \cdots & X_{1p}\\ X_{21} & X_{22} & \cdots & X_{2p}\\ \vdots & \vdots & \ddots & \vdots \\ X_{n1} & X_{n2} & \cdots & X_{np} \end{array}\right]}_{n\times p}=\left(X_{1},X_{2},\cdots ,X_{p}\right)$$

(3) $$\alpha ={\left(\begin{array}{c} \alpha \\ \alpha \\ \vdots \\ \alpha \end{array}\right)}_{n\times 1,}\beta ={\left(\begin{array}{c} \beta \\ \beta \\ \vdots \\ \beta \end{array}\right)}_{p\times 1,}\mu ={\left(\begin{array}{c} \mu \\ \mu \\ \vdots \\ \mu \end{array}\right)}_{n\times 1}$$

Ordinary least squares is used to estimate the unknown parameters, but usually, in real data, multi-collinearity is common; the determinant of the correlation matrix of the independent variables will be approximately zero; in other words, *X*^*T*^*X* is singular, which can lead to inaccurate least squares estimates or inaccurate parameter estimates. The singularity of the *X*^*T*^*X*+*KI* regression coefficients is improved by *X*^*T*^*X* adding the normal unit *KI* matrix, which is then used $\hat{B}\left(k\right)\text{=}{\left(X^{T}X+KI\right)}^{\text{-}1}X^{T}Y$ as an estimate of the regression coefficients, a value that is more stable than the least squares estimate and $\hat{B}\left(k\right)$ is known as the ridge estimate of the regression coefficients.

### Reliability and Validity Analysis

The reliability can be reflected by calculating the Cronbach's alpha value of each scale, and SPSS25.0 was applied in this research. As shown in Table 1, the Alpha value of the Entrepreneurship Course Scale is 0.888, the Alpha value of the Entrepreneurship Faculty Scale is 0.918, the Alpha value of the Entrepreneurship Competition Scale is 0.910, the Alpha value of the Entrepreneurship Practice Scale is 0.958, the Alpha value of the Entrepreneurship Policy Scale is 0.961 and the Alpha value of the Entrepreneurship Education Performance Scale is 0.969, all of which are >0.8, demonstrating that the reliability of the scales is good. The test of validity can be conducted first by exploratory factor analysis using SPSS25.0, and the results are shown in Table 1. The KMO values of 0.731 for entrepreneurship course, 0.754 for entrepreneurship faculty, 0.752 for entrepreneurship competition, 0.920 for entrepreneurship practice, 0.864 for entrepreneurship policy, and 0.906 for entrepreneurship education performance are all higher than 0.7, indicating a strong bias correlation of the variables, while the calculated chi-square values of Bartlett's statistic for each scale are all significant, indicating that the correlation coefficient matrix between the variables is unlikely to be a unit array, and there is a correlation between them; the correspondence between the question items of each scale and the scale formation factors is consistent with the study's expectation, and the factor loading values are all above 0.5, with no cross-factor phenomenon.

**Table 1:** Cronbach's alpha values and exploratory factor analysis.

**Scale**	**Title item**	**Cronbach's alpha value**	**KMO values**	**Bartlett's spherical test**	**Factor load value**
Entrepreneurship course	Diverse types of entrepreneurship education course	0.888	0.731	43609.678^***^	0.873
	The content of the entrepreneurship course is closely integrated with your own professional knowledge				0.915
	The content of the entrepreneurship course is closely aligned with the cutting-edge trends of the times				0.924
Entrepreneurship faculty	Teachers teach a variety of styles	0.918	0.754	54033.388^***^	0.912
	Teachers with entrepreneurial experience				0.930
	Teachers with extensive experience in teaching entrepreneurship education				0.937
Entrepreneurship competition	Variety of entrepreneurship competition	0.910	0.752	50644.743^***^	0.923
	Entrepreneurship competition projects entered are more likely to be landed				0.932
	High degree of integration of entrepreneurship competition projects with the profession				0.907
Entrepreneurship practice	Entrepreneurship practice with on and off-campus mentors	0.958	0.920	164087.734^***^	0.877
	Entrepreneurship practice is supported by a dedicated start-up fund				0.895
	The school offers an integrated entrepreneurship practice service				0.934
	There is an independent college students pioneer park for entrepreneurship practice				0.906
	Dedicated off-campus practice base for entrepreneurship practice				0.923
	High degree of integration of practical entrepreneurship projects with professional studies				0.918
Entrepreneurship policy	State tax relief for university students starting their own businesses	0.961	0.864	116717.870^***^	0.946
	Local governments simplify the application process for university student business registration				0.955
	The university provides a start-up fund (interest-free loan) for starting a business				0.946
	Free training from the community to guide your business				0.938
Entrepreneurship education performance	Enriching entrepreneurial knowledge	0.969	0.906	170266.543^***^	0.952
	Cultivating innovation spirit				0.953
	Improving entrepreneurial skills				0.958
	Stimulating entrepreneurial intentions				0.955
	Overall satisfaction				0.900

*N = 24677.*

*** *p < 0.01*.

The validation factor analysis was then used to further explore the reliability and validity, and the results obtained using the AMOS 24.0 software are shown in Table 2. The standardized factor loadings for the three measures of entrepreneurship course were 0.831, 0.849, and 0.884; the standardized factor loadings for the three measures of entrepreneurship faculty were 0.867, 0.895, and 0.905; the standardized factor loadings for the three measures of entrepreneurship competition were 0.893, 0.885, and 0.861; the standardized factor loadings for the six measures of entrepreneurship practice were 0.848, 0.865, 0.925, 0.884, 0.909, and 0.909; the standardized factor loadings for the four measures of entrepreneurship policy were 0.930, 0.944, 0.924, and 0.913; the standardized factor loadings for the five measures of entrepreneurship education performance were 0.945, 0.946, 0.947, 0.944, and 0.869. All standardized factor loadings were significant (the first question item for each factor did not report significance). This indicates a strong relationship between the measurement terms and the factors. The software also outputs overall model fit indices, including absolute fit index, value-added fit index and simplicity fit index, with absolute fit index: RMR = 0.031 (<0.05), RMSEA = 0.077 (<0.08), GFI = 0.887 (≈0.9), AGFI = 0.857 (≈0.9); value-added fitness index: CFI = 0.955 (>0.9), NFI = 0.955 (>0.9), TLI (NNFI) = 0.948 (>0.9), IFI = 0.955 (>0.9), RFI = 0.948 (>0.9); simplicity fit index: PGFI = 0.701 (>0.5), PNFI = 0.820 (>0.5), PCFI = 0.820 (>0.5). Notably, most of the fitness indicators of the validated factor analysis model meet the criteria. The two indicators of GFI and AGFI were <0.9, but close to 0.9. The purpose of the analysis is to validate the validity of these indicators, which is of very low concern, therefore the model effect is acceptable. Using standardized factor loadings, composite reliability (CR) can be calculated as an indicator of the reliability of the latent variables, with composite reliability of 0.891 for entrepreneurship course, 0.919 for entrepreneurship faculty, 0.911 for entrepreneurship competition, 0.958 for entrepreneurship practice, 0.961 for entrepreneurship policy, and 0.970 for entrepreneurship education performance. All of these values are >0.7 standard and have good reliability. The average variance extracted (AVE)—an indicator of convergent validity, can also be calculated using the standardized factor loadings, with an AVE of 0.731 for entrepreneurship course, 0.791 for entrepreneurship faculty, 0.774 for entrepreneurship competition, 0.793 for entrepreneurship practice, and 0.861 for entrepreneurship policy, and 0.866 for entrepreneurship education performance. All of which are greater than the standard of 0.5, and have good convergent validity.

**Table 2:** Validation factor analysis.

**Factor (latent variable)**	**Measurement items (Explicit variable)**	**Standardized factor loading values**	***P***	**Composite reliability (CR)**	**Average variance extracted (AVE)**
Entrepreneurship course	Diverse types of entrepreneurship education course	0.831	–	0.891	0.731
	The content of the entrepreneurship course is closely integrated with your own professional knowledge	0.849^***^	0.000		
	The content of the entrepreneurship course is closely aligned with the cutting-edge trends of the times	0.884^***^	0.000		
Entrepreneurship faculty	Teachers teach a variety of styles	0.867	–	0.919	0.791
	Teachers with entrepreneurial experience	0.895^***^	0.000		
	Teachers with extensive experience in teaching entrepreneurship education	0.905^***^	0.000		
Entrepreneurship competition	Variety of entrepreneurship competition	0.893	–	0.911	0.774
	Entrepreneurship competition projects entered are more likely to be landed	0.885^***^	0.000		
	High degree of integration of entrepreneurship competition projects with the profession	0.861^***^	0.000		
Entrepreneurship practice	Entrepreneurship practice with on and off-campus mentors	0.848	–	0.958	0.793
	Entrepreneurship practice is supported by a dedicated start-up fund	0.865^***^	0.000		
	The school offers an integrated entrepreneurship practice service	0.925^***^	0.000		
	There is an independent college students pioneer park for entrepreneurship practice	0.884^***^	0.000		
	Dedicated off-campus practice base for entrepreneurship practice	0.909^***^	0.000		
	High degree of integration of practical entrepreneurship projects with professional studies	0.909^***^	0.000		
Entrepreneurship policy	State tax relief for university students starting their own businesses	0.930	–	0.961	0.861
	Local governments simplify the application process for university student business registration	0.944^***^	0.000		
	The university provides a start-up fund (interest-free loan) for starting a business	0.924^***^	0.000		
	Free training from the community to guide your business	0.913^***^	0.000		
Entrepreneurship education performance	Enriching entrepreneurial knowledge	0.945	–	0.970	0.866
	Cultivating innovation spirit	0.946^***^	0.000		
	Improving entrepreneurial skills	0.947^***^	0.000		
	Stimulating entrepreneurial intentions	0.944^***^	0.000		
	Overall satisfaction	0.869^***^	0.000		

*N = 24677.*

*** *p < 0.01*.

In summary, the reliability and validity of the study scales were good.

### Common Method Bias Test

Common method variance is the use of the same measurement tool that can lead to spurious common variation among traits, and the bias generated by common method variance is called common method bias (CMB), which can affect the accuracy of the study results. The CMB was tested by controlling for non-measurable potential method factors. Based on the above validated factor analysis model, a new model was built by using all question items as indicators of method factors and outputting the relevant indicators with absolute fit index: RMR = 0.073 (<0.05), RMSEA = 0.073 (<0.08), GFI = 0.899 (≈0.9), AGFI = 0.869 (≈0.9); value-added fit index: CFI = 0.961 (>0.9), NFI = 0.960 (>0.9), TLI (NNFI) = 0.953 (>0.9), IFI = 0.961 (>0.9), RFI = 0.953 (>0.9), and simplicity fit index: PGFI = 0.693 (>0.5), PNFI = 0.804 (>0.5), PCFI = 0.804 (>0.5). The magnitude of change from the original model is not significant; therefore, there is no serious CMB in this study.

## Analysis of the Research Process and Results

### Descriptive Statistical Analysis

The number of male medical students in this study was 6,338 (25.7%), and the number of female students was 18,339 (74.3%). They came from 30 provinces (autonomous regions and municipalities) in China, with 4,507 from Henan, the highest percentage at 18.3%. Shanxi (3,580), Heilongjiang (3,399), and Fujian (3,036) followed closely behind, accounting for over 10% of the total. The number of survey respondents whose parents (or other immediate family members) had experience in setting up businesses was 5,005 or 20.3%.

Descriptive statistical analysis of the scale items is presented in Table 3. The mean value of the five questions on the performance of entrepreneurship education for medical students was >3.6, indicating that medical students are currently more inclined to “relatively agree” with entrepreneurship education performance in higher education.

**Table 3:** Descriptive statistics.

**Scale**	**Title item**	**Minimum value**	**Maximum value**	**Average value**	**Standard deviation**
Entrepreneurship course	Diverse types of entrepreneurship education course	1	5	3.334	1.000
	The content of the entrepreneurship course is closely integrated with your own professional knowledge	1	5	3.254	1.036
	The content of the entrepreneurship course is closely aligned with the cutting-edge trends of the times	1	5	3.438	0.972
Entrepreneurship faculty	Teachers teach a variety of styles	1	5	3.482	0.969
	Teachers with entrepreneurial experience	1	5	3.335	0.991
	Teachers with extensive experience in teaching entrepreneurship education	1	5	3.483	0.991
Entrepreneurship competition	Variety of entrepreneurship competition	1	5	3.433	0.979
	Entrepreneurship competition projects entered are more likely to be landed	1	5	3.279	0.975
	High degree of integration of entrepreneurship competition projects with the profession	1	5	3.337	0.985
Entrepreneurship practice	Entrepreneurship practice with on and off-campus mentors	1	5	3.574	0.948
	Entrepreneurship practice is supported by a dedicated start-up fund	1	5	3.506	0.971
	The school offers an integrated entrepreneurship practice service	1	5	3.445	0.962
	There is an independent college students pioneer park for entrepreneurship practice	1	5	3.455	1.009
	Dedicated off-campus practice base for entrepreneurship practice	1	5	3.390	1.001
	High degree of integration of practical entrepreneurship projects with professional studies	1	5	3.430	0.976
Entrepreneurship policy	State tax relief for university students starting their own businesses	1	5	3.615	0.922
	Local governments simplify the application process for university student business registration	1	5	3.594	0.922
	The university provides a start-up fund (interest-free loan) for starting a business	1	5	3.574	0.944
	Free training from the community to guide your business	1	5	3.547	0.956
Entrepreneurship education performance	Enriching entrepreneurial knowledge	1	5	3.720	0.892
	Cultivating innovation spirit	1	5	3.733	0.895
	Improving entrepreneurial skills	1	5	3.733	0.889
	Stimulating entrepreneurial intentions	1	5	3.732	0.887
	Overall satisfaction	1	5	3.630	0.919

*N = 24677*.

The mean scores for the questions related to course, faculty, competition, practice, and policy ranged from 3.254 to 3.615. The highest mean score for entrepreneurship course was “The content of the entrepreneurship course is closely aligned with the cutting-edge trends of the times,” while the lowest mean score was “The content of the entrepreneurship course is closely integrated with your own professional knowledge.” The highest mean item for entrepreneurship faculty was “Teachers with extensive experience in teaching entrepreneurship education,” while the lowest mean item was “Teachers with entrepreneurial experience.” The highest mean item for entrepreneurship competition was “Variety of entrepreneurship competition,” while the lowest mean item was “Entrepreneurship competition projects entered are more likely to be landed.” The highest mean item in entrepreneurship practice was “Entrepreneurship practice with on and off-campus mentors,” while the lowest mean item was “Dedicated off-campus practice base for Entrepreneurship practice.” The highest mean item in entrepreneurship policy was “State tax relief for university students starting their own businesses,” while the lowest mean item was “Free training from the community to guide your business.”

### Ridge Regression Analysis

**Figure 2:**
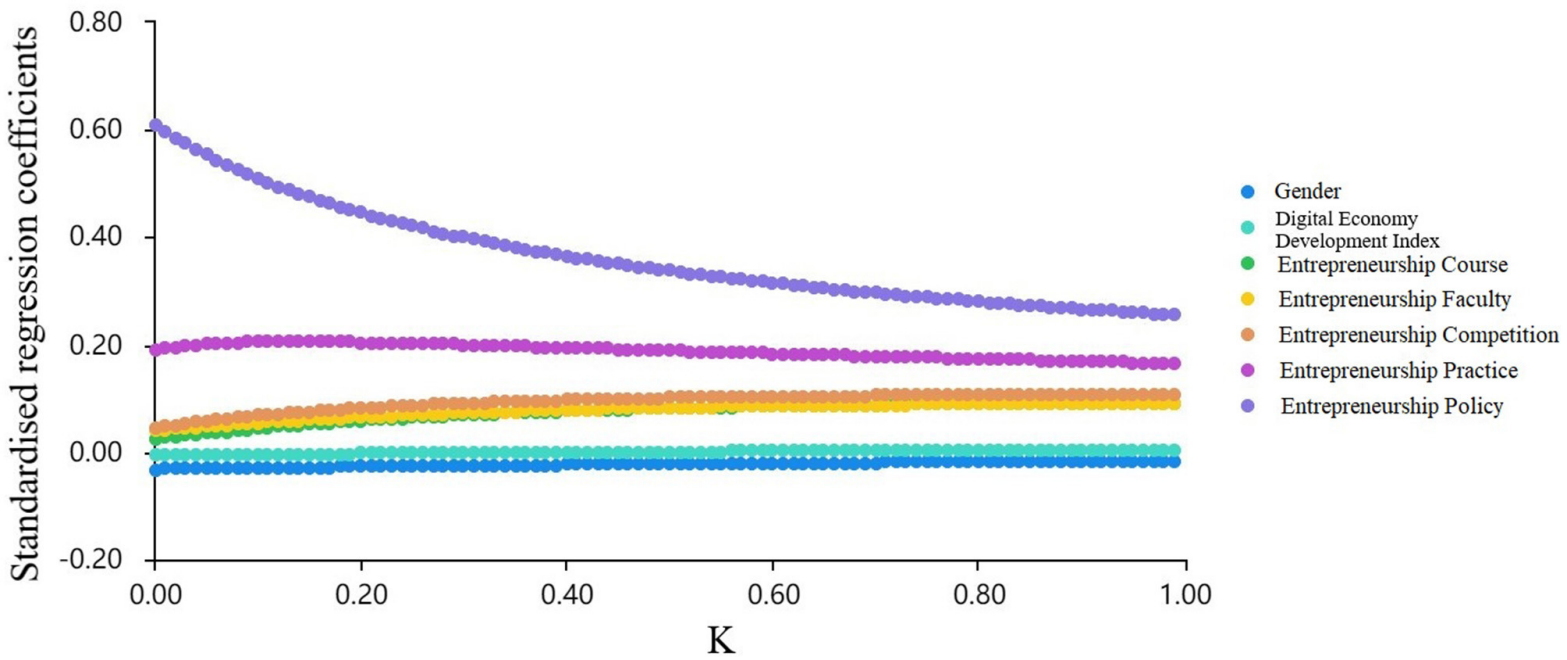
Ridge trace plot.

Ridge regression can be calculated using ridge regression technique, a set of macro programs in SPSS. It is appropriate because it can improve the consistency of parameter estimates compared to ordinary linear regression and to obtain more realistic and reliable results for the regression parameters. The *K-*value needs to be confirmed in conjunction with the ridge trace plot before the ridge regression analysis. The principle of *K-*value selection is the minimum *K-*value when the standardized regression coefficient of each independent variable tends to be stable. Gender, digital economy development index, entrepreneurship course, entrepreneurship faculty, entrepreneurship competition, entrepreneurship practice, and entrepreneurship policy were used as independent variables, and entrepreneurship education performance was used as the dependent variable to conduct a ridge regression analysis using SPSS software. A ridge trace plot is shown in Figure 2. When the *K-*value was 0.99, the standardized regression coefficient of the independent variable tended to be stable at this point. Therefore, the optimum *K-*value was set at 0.99.

Once the *K-*values are determined, they can be entered. The ridge regression model estimates were derived from the software, and the results are presented in Table 4.

**Table 4:** Results of the ridge regression analysis.

**Independent variable**	**Non-standardized coefficients**		**Standardization factor**	***t***	***p***	***R*** **^2^**	***F***
	**Coefficient values**	**Standard error**	**Coefficient values**				
Constants	−0.002	0.007	–	−0.221	0.825	0.651	*F*_(7,24,669)_ = 6581.572, *p* = 0.000
Gender	−0.038^***^	0.004	−0.017^***^	−8.864	0.000		
Digital economy development index	0.0003^**^	0.0002	0.004^**^	1.987	0.047		
Entrepreneurship course	0.092^***^	0.001	0.092^***^	70.655	0.000		
Entrepreneurship faculty	0.091^***^	0.001	0.091^***^	61.624	0.000		
Entrepreneurship competition	0.106^***^	0.001	0.106^***^	79.813	0.000		
Entrepreneurship practice	0.165^***^	0.001	0.165^***^	119.396	0.000		
Entrepreneurship policy	0.256^***^	0.002	0.256^***^	158.227	0.000		

*N = 24677.*

** *p < 0.05;*

*** *p < 0.01. Dependent variable: entrepreneurship education performance*.

From Table 4, gender, digital economy development index, entrepreneurship course, entrepreneurship faculty, entrepreneurship competition, entrepreneurship practice, and entrepreneurship policy were used as independent variables, while entrepreneurship education performance was used as the dependent variable for the ridge regression analysis. The *K-*value was set at 0.99. Notably, the model R-squared value was 0.651, implying that gender, digital economy development index, entrepreneurship course, entrepreneurship faculty, entrepreneurship competition, entrepreneurship practice, and entrepreneurship policy could explain 65.1% of the variation in entrepreneurship education performance. The model passed the *F*-test (*F* = 6581.572, *p* = 0.000 < 0.01).

The standardized coefficient of gender was −0.017 (*t* = −8.864, *p* = 0.000 < 0.01), implying gender has a significant negative relationship with entrepreneurship education performance. The standardized coefficient of the digital economy development index was 0.004 (*t* = 1.987, *p* = 0.047 < 0.05), implying that the digital economic development index has a significant positive relationship with entrepreneurship education performance. The standardized coefficient for entrepreneurship course was 0.092 (*t* = 70.655, *p* = 0.000 < 0.01), implying that entrepreneurship course would have a significant positive impact on entrepreneurship education performance. The standardized coefficient for entrepreneurship faculty was 0.091 (*t* = 61.624, *p* = 0.000 < 0.01), implying that entrepreneurship faculty would have a significant positive relationship with entrepreneurship education performance. The standardized coefficient for entrepreneurship competition was 0.106 (*t* = 79.813, *p* = 0.000 < 0.01), implying that entrepreneurship competition would have a significant positive influence on entrepreneurship education performance. The standardized coefficient for entrepreneurial practice was 0.165 (*t* = 119.396, *p* = 0.000 < 0.01), implying that entrepreneurial practice has a significant positive relationship with entrepreneurship education performance. The standardized coefficient for entrepreneurship policy was 0.256 (*t* = 158.227, *p* = 0.000 < 0.01), implying that entrepreneurship policy would have a significant positive influence on entrepreneurship education performance.

## Discussion

This research theoretically explored the relationship between gender, digital economy, entrepreneurship course, entrepreneurship faculty, entrepreneurship competition, entrepreneurship practice, entrepreneurship policy, and entrepreneurship education performance, and proposed the EEPMS theoretical model. Based on the data of 24,677 research questionnaires, the relationship between them was studied empirically using ridge regression. This study answers three questions raised at the beginning of the article. That is, there are differences in the entrepreneurship education performance among medical students of different genders; the digital economy impacts on EEPMS; entrepreneurship course, entrepreneurship faculty, entrepreneurship competition, entrepreneurship practice, and entrepreneurship policy drives the EEPMS, and the impact of entrepreneurship policy is the most obvious, followed by entrepreneurship practice and entrepreneurship competition, followed by entrepreneurship course and entrepreneurship faculty.

The gender differences between men and women will lead to differences in their style of doing things, also reflected in the EEPMS. Females are interested in learning more entrepreneurship knowledge and skills. Entrepreneurship education will help females to eliminate entrepreneurship barriers and obtain higher entrepreneurship education performance (Higgins et al., 2018). The results of our research show that the entrepreneurship education performance of female medical students is higher—consistent with the analysis mentioned in the literature review that women are more able to take care of others and their more humane characteristics will give them more advantages of the medical and health care field (Hechavarría et al., 2017; Dickel and Eckardt, 2021). However, in the current entrepreneurship education of medical students, it is rarely carried out by gender. Paying attention to gender differences does not imply gender inequality. Contrarily, it is based on the concept of gender equality to truly realize that both men and women have the right to receive entrepreneurship education. Full consideration should be given to the characteristics of men and women so that they can play to their strengths in specific situations. The purpose and effect of entrepreneurship education are better understood.

We have entered the era of the digital economy. The digital facilities construction, digital technology application, and digital industries development are changing existing economic operations, development methods, and people's production and lifestyles. Digital economy has brought changes to entrepreneurial activities and processes (Srinivasan and Venkatraman, 2018), and reduced entrepreneurship cost (Zhao et al., 2015), which can promote entrepreneurial behavior and results (Mack et al., 2017). However, there is little research on whether digital economy can improve entrepreneurship education performance. This study provides evidence that digital economy has a positive impact on entrepreneurship education performance of medical students. The digital economy era provides a new teaching method and education platform for entrepreneurship, allowing students with limited learning resources to break through the limitations of space and enhance students' enthusiasm for participating in entrepreneurship education through network interaction and virtual entrepreneurial incubation platforms. Entrepreneurship education should also adapt to the various encounters and challenges brought to us by the digital economy era, and make corresponding adjustments to entrepreneurial education to adapt to development.

Entrepreneurship policy is oriented and supportive, which not only leads universities to implement entrepreneurship education properly but also clears the obstacles for college students in entrepreneurship by establishing a mechanism to support entrepreneurship funds, simplifying the business registration process, and a mechanism to transform project technology (Kang and Xiong, 2021). Thus, it improves students' willingness to start a business, makes them more active in entrepreneurship teaching, and improves the quality of entrepreneurship education (Huang et al., 2021).

Theoretical and practical learning are two necessary paths for students' entrepreneurial activity knowledge. The theory is inseparable from practice, and practice is inseparable from theory. On the one hand, Entrepreneurship course provides students with an opportunity to deepen their learning, through which they can master the basic theoretical knowledge and skills to lay a solid foundation for future entrepreneurial practice activities (Foster and Lin, 2003). Then again, students can better understand what they have learned by participating in entrepreneurship competitions and activities (Wen and Chen, 2007; Wang, 2020). As the Chinese proverb goes, “it is better to read 10,000 books than to travel 10,000 miles.” Through practice, students can be better brought into the entrepreneurial scenario so that they can experience the opportunities and challenges in the process of entrepreneurship in advance and enhance their entrepreneurial ability. Entrepreneurship faculty is a transmitter of entrepreneurship education knowledge, and entrepreneurial knowledge, teaching ability, and entrepreneurial experience impact the entrepreneurship education process (Ruskovaara and Pihkala, 2013). Teachers with strong teaching abilities can fully transfer their knowledge to students, and at the same time, they can also combine their entrepreneurial experience to improve the practicality of the knowledge taught in the classroom (Fayolle, 2013). Moreover, teachers who teach by sharing their entrepreneurial experience break through the boring and playful sense of traditional theory, making the entrepreneurship course more vivid and concrete, and ensuring the quality of the entrepreneurship education course (Bechard and Gregoire, 2005).

## Conclusion

The research in this article provides valuable insights into the EEPMS, supporting hypotheses H1, H2, H3, H4, H5, H6, and H7. Gender has a significant effect on the EEPMS, and the digital economy, entrepreneurship course, entrepreneurship faculty, entrepreneurship competition, entrepreneurship practice, and entrepreneurship policy have a significant positive effect on the EEPMS. The results of the verification of the research hypothesis are presented in Table 5.

**Table 5:** Research hypothesis.

	**Research hypothesis**	**Result**
H1	Gender significantly affects the entrepreneurship education performance of medical students.	Accept
H2	The digital economy has a significant positive impact on the entrepreneurship education performance of medical students.	Accept
H3	Entrepreneurship course has a significant positive impact on the entrepreneurship education performance of medical students.	Accept
H4	Entrepreneurship faculty has a significant positive impact on the entrepreneurship education performance of medical students.	Accept
H5	Entrepreneurship competition has a significant positive impact on the entrepreneurship education performance of medical students.	Accept
H6	Entrepreneurship practice has a significant positive impact on the entrepreneurship education performance of medical students.	Accept
H7	Entrepreneurship policy has a significant positive impact on the entrepreneurship education performance of medical students.	Accept

### Implications for Theory

This study makes three theoretical contributions to the research on the driving factors of entrepreneurial education performance of medical students in the digital age.

First, the study initiatively constructed a research model (EEPMS model) that incorporates the effects of gender, digital economy, entrepreneurship course, entrepreneurship faculty, entrepreneurship competition, entrepreneurship practice, and entrepreneurship policy on entrepreneurship education performance of medical students. As the primary force to promote social innovation, medical students have strong learning ability, professional theory, and knowledge, and can transform entrepreneurial ideas into entrepreneurial behavior (Zsuzsoka and Imogen, 2017). Combined with the EEPMS theoretical model, this study explores the multiple factors affecting the entrepreneurship education performance of medical students, further improves the theoretical understanding, and provides some inspiration and reference for follow-up research and practical policies.

Second, the study discusses the EEPMS from a new perspective of digital economy. The advent of digital age and the development of the digital technology has brought profound changes to the whole society, and at the same time, it has also brought many opportunities and challenges to entrepreneurship education (Giones and Brem, 2017; Srinivasan and Venkatraman, 2018). Therefore, it is of great theoretical significance to explore the impact of the digital economy on entrepreneurship education performance of medical students. Construct the theoretical framework of digital economy and entrepreneurship education, combine the digital economy with the EEPMS, closely follow the background of the times, and further enrich the theoretical understanding of the EEPMS in the digital era.

Third, the study innovatively uses new method that ridge regression model to analyze empirically the performance drivers of EEPMS in the digital era with large sample data, which covers 30 provinces (autonomous regions and municipalities directly under the Central Government) in China, and collects 24,677 questionnaires. The ridge regression model is used to analyze empirically the performance drivers of EEPMS in the digital era to obtain a more comprehensive and robust rational understanding.

### Implications for Practice

This study puts forward four practical implications for research on the performance drivers of entrepreneurship education for medical students in the digital era.

#### Create a New Model of “Digital + Entrepreneurship Education of Medical Students” Against the Background of the Digital Economy Era

Big data, artificial intelligence, Internet technology have promoted the continuous transformation of the entrepreneurial mode, which produces great entrepreneurial opportunities and entrepreneurship education reform (Zhao et al., 2015; Srinivasan and Venkatraman, 2018). Especially since COVID-19, the rapid development of online education has accelerated the digital transformation of education, in which digital technology plays an important role (Daniela et al., 2021; Slišāne et al., 2021). In the digital age, knowledge is rich and fast updating, which requires entrepreneurship course to meet the diversified needs of learners (Wu et al., 2019). Digital technology combines with a wide range of resources to create an effective Online learning environment (Kop, 2011), and creates a Massive Open Online Course (MOOC) platform. Learners can learn a variety of entrepreneurship course through MOOC that discuss entrepreneurship issues from different perspectives, provide good entrepreneurship guidance and promote the development of entrepreneurship education (Wu et al., 2019). The Digital is a major trend in the development of the times. With the educational concept of “Digital + Medical Entrepreneurship Education,” we can expand the application model of Digital Medical Education by building online cloud classrooms, virtual practice platforms, cloud service entrepreneurial knowledge databases, and other platforms. It can overcome the limitations of time, space, and funds, improve students' sense of experience in entrepreneurship education, and promote entrepreneurship education performance in colleges and universities. Moreover, medical students' entrepreneurship education is often inseparable from medical experiments. Through the digital immersive platform construction, we can use this platform to transform abstract and complex medical functional experiments into a visible and perceptible practical process, greatly increasing the interest and efficiency, which is helpful for the development and performance improvement of medical entrepreneurship education (Kesner et al., 2018).

#### Increase Policy Support Efforts to Improve Relevant Institutional Mechanisms Continuously

With the outstanding contribution of entrepreneurship education to national development, an increasing number of universities have made entrepreneurship education a priority area of development and established the corresponding matching to policy systems to improve and develop entrepreneurship education continuously. In the eighties, because of the outbreak of the oil crisis, the British economy fell into a negative growth mode, and faced with many unemployed groups, the British government passed a designated act to strengthen the cooperation between universities and industry and business, and then in 1987, the British government officially launched a project called “Higher Education Entrepreneurship” (Whiteley, 1995). This has helped to clarify and visualize entrepreneurship education and guide the entrepreneurship education implementation in universities. At present, although more and more policies have been formulated and issued, but many countries, especially developing countries, still have problems that entrepreneurship policies are imperfect, inadequate, and insufficient (Yan, 2020). With the high degree specialization of medical, medical students often have a greater demand for financing and finance funds to start their businesses (Douglas and Shepherd, 2002), and that often requires policy to achieve (Audretsch, 2015; Kang and Xiong, 2021). And, government should provide more convenient examination and approval processes and more professional social training.

#### Strengthen the Concept of “Knowledge-Action Unity” Education and Promote the Organic Integration of Course and Practice

The theory is applied to practice and condensed into a theory in practice, and the two complement each other. Entrepreneurship education course learning and entrepreneurship education practice learning are indispensable (Foster and Lin, 2003; Higgins et al., 2018; Otache et al., 2020). For the cultivation of entrepreneurship education for medical students, we should cultivate the “medical + entrepreneurial” thinking, and let students experience the actual training process of entrepreneurship. Forming a “knowledge-action unity” entrepreneurship education system through equipment support, entrepreneurial incubation base, entrepreneurial competition, project investment, and so on. Thus, to increase the enthusiasm and reality of students to participate in entrepreneurship education and reduce the risk of entrepreneurship in the future. Medical students' entrepreneurship education should integrated into specialty to construct an entrepreneurship education model with characteristics of medical colleges and universities from the aspects of curriculum content, teaching method, faculty, practice, and so on. Besides, it is also very important to build a three-level platform for medical innovation, R&D, and practice platform of “university -college-Laboratory” to enhance the cooperation and contact between schools and companies (Dahlstedt and Hertzberg, 2012). Additionally, it is important to expand industry-university cooperation platforms such as medical science and technology cities and medical health towns outside the school.

#### View Gender Differences Correctly and Promote Joint Development Among Male and Female Students

Gender differences in entrepreneurship between men and women are undeniable, but men and women also have their advantages in the process of entrepreneurship. Men are more adventurous and more willing to accept adjustable entrepreneurial models. Compared to men, women are more delicate, rigorous, and kind, therefore it is also easier to have a good relationship with team members in a harmonious way. Based on this, we should establish the concept of gender equality, correctly treat the gender differences between male and female students, and give full play to the maximum advantages of each in the process of entrepreneurship education (Zisser et al., 2019).

## Limitations and Further Research Opportunities

This research explores the digital perspective in exploring the EEPMS, explores the driving factors of EEPMS in the digital age, clarifies each factor's importance, and proposes solutions to improve the EEPMS. However, since research on the digital economy has just started, the literature and data are insufficient, and the specific mechanism research is insufficient. Of course, this also provides a fresh perspective for later research: perhaps it is possible to conduct in-depth research on the digital entrepreneurship of medical students. The driving factors in this paper include entrepreneurship course, entrepreneurship faculty, entrepreneurship competition, entrepreneurship practice and entrepreneurship policy, and the influencing factors of digital economy and gender. There are other factors that may drive the entrepreneurship education performance of medical students, but other factors are not considered in this study due to the availability of research. We aim to be more comprehensive in future studies. Another limitation of this study is that although the data in this article have a large sample size, a wide range of surveys, and a certain degree of representativeness, the data used are cross-sectional data, and heterogeneity cannot be observed. Future research can strengthen the collection of time series tracking data to better understand the EEPMS.

## Data Availability Statement

The original contributions presented in the study are included in the article, further inquiries can be directed to the corresponding authors.

## Author Contributions

ZL and GZ described and developed the review and the hypothesis. ZL, MZ, and ZH was involved in the data collection process. ZL and JW performed the analysis, interpretation of the results and formulated the main conclusions. LZ conducted the questionnaire survey and editing. SZ and ZH formulated the study limitations and future directions for research. LZ conducted the questionnaire survey and editing. All authors helped in editing and formatting the paper.

## Funding

This work was supported by the key project of the National Social Science Fund of China–the Research on Barriers and Policy Support Mechanisms for Female Entrepreneurship in the Digital Era (20ASH012).

## Conflict of Interest

The authors declare that the research was conducted in the absence of any commercial or financial relationships that could be construed as a potential conflict of interest.

## Publisher's Note

All claims expressed in this article are solely those of the authors and do not necessarily represent those of their affiliated organizations, or those of the publisher, the editors and the reviewers. Any product that may be evaluated in this article, or claim that may be made by its manufacturer, is not guaranteed or endorsed by the publisher.
